# Dispiro­[cyclo­propane-1,5′-*endo*-tricyclo­[5.2.1.0^2,6^]deca-3,8-diene-10′,1′′-cyclo­propane]

**DOI:** 10.1107/S1600536810021951

**Published:** 2010-06-16

**Authors:** Rafał Grubba, Łukasz Ponikiewski, Jerzy Pikies

**Affiliations:** a Chemical Faculty, Gdańsk University of Technology, Narutowicza 11/12, Gdańsk PL 80233, Poland

## Abstract

The title compound, C_14_H_16_, is built up from three five-membered rings. Two of the five-membered rings display an envelope conformation and the third one is almost planar (r.m.s. deviation = 0.014 Å).

## Related literature

For the synthesis, see: Khusnutdinov *et al.* (1988 ▶); Wilcox *et al.* (1961 ▶). For related structures, see: Caira *et al.* (1995 ▶); Haumann *et al.* (1997 ▶); Brookings *et al.* (2001 ▶).
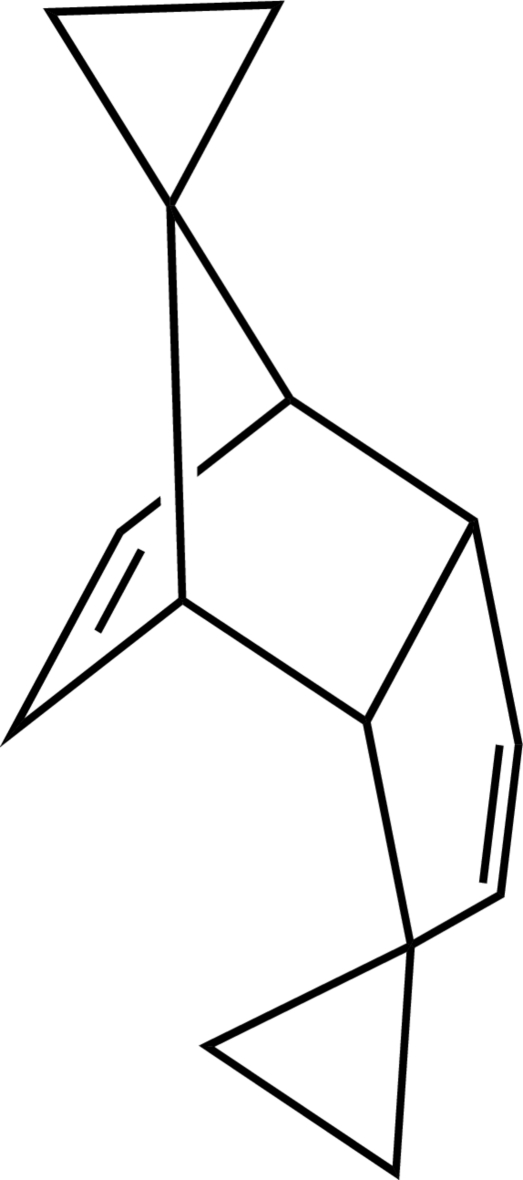


## Experimental

### 

#### Crystal data

C_14_H_16_
*M* *_r_* = 184.27
Triclinic, 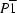

*a* = 6.4079 (5) Å
*b* = 8.6355 (8) Å
*c* = 10.7216 (10) Å
α = 68.488 (9)°
β = 81.625 (7)°
γ = 73.351 (8)°
*V* = 528.27 (8) Å^3^
*Z* = 2
Mo *K*α radiation
μ = 0.07 mm^−1^
*T* = 293 K
0.23 × 0.22 × 0.21 mm

#### Data collection

Oxford Diffraction Xcalibur S diffractometer
Absorption correction: multi-scan (*CrysAlis PRO*; Oxford Diffraction, 2009 ▶) *T*_min_ = 0.775, *T*_max_ = 1
3444 measured reflections
2269 independent reflections
1348 reflections with *I* > 2σ(*I*)
*R*_int_ = 0.024

#### Refinement

*R*[*F* ^2^ > 2σ(*F* ^2^)] = 0.060
*wR*(*F* ^2^) = 0.168
*S* = 0.99
2269 reflections
127 parameters
H-atom parameters constrained
Δρ_max_ = 0.22 e Å^−3^
Δρ_min_ = −0.15 e Å^−3^

### 

Data collection: *CrysAlis PRO* (Oxford Diffraction, 2009 ▶); cell refinement: *CrysAlis PRO*; data reduction: *CrysAlis PRO*; program(s) used to solve structure: *SHELXS97* (Sheldrick, 2008 ▶); program(s) used to refine structure: *SHELXL97* (Sheldrick, 2008 ▶); molecular graphics: *ORTEP-3 for Windows* (Farrugia, 1997 ▶); software used to prepare material for publication: *WinGX* (Farrugia, 1999 ▶).

## Supplementary Material

Crystal structure: contains datablocks I, global. DOI: 10.1107/S1600536810021951/gk2280sup1.cif

Structure factors: contains datablocks I. DOI: 10.1107/S1600536810021951/gk2280Isup2.hkl

Additional supplementary materials:  crystallographic information; 3D view; checkCIF report

## Figures and Tables

**Fig. 1.**
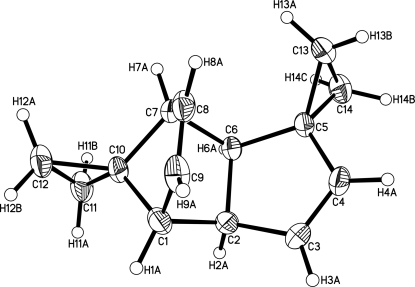
The molecular structure of the title compound showing the atom-labelling scheme and displacement ellipsoids at the 25% probability level.

## supplementary crystallographic information

### Comment

The title compound (I) is a product of cyclodimerization of spiro[2.4]hepta-4,6-diene. After few weeks of storing of the starting diene at room temperature big crystals of (I) were isolated with relatively high yield. In contrast to previously reported method of synthesis of (I) (Khusnutdinov *et al.* 1988), we did not use the additional heating and the catalyst.

The X-ray crystallographic analysis confirms this proposed molecular structure (Fig. 1). The C_14_H_16_ is built up from three five-membered rings and two three-membered rings. The one of the five-membered rings (C2—C3—C4—C5—C6) is almost planar. The mean deviation of the five atoms C2, C3, C4, C5, C6 from their least-squares plane is 0.0136 Å. Additionally, the C5 atom is a junction between the five-membered ring and a cyclopropane ring. The dihedral angle between the central ring planes is 89.89 (2)°.

The second and third five-membered rings (C1—C2—C6—C7—C10 and C7—C8—C9—C1—C10) have an envelope conformation.The C10 atom is a junction with the second cyclopropane ring.

The typical C2=C3 and C6=C7 double bonds lengths 1.312 (3) Å, 1.309 (3) Å respectively suggest that the C2, C3, C6, C7 atoms are *sp*^2^ hybridized. The bond lengths and angles are within normal ranges (Brookings *et al.* 2001; Caira *et al.* 1995; Haumann *et al.* 1997).

### Experimental

Spiro[2.4]hepta-4,6-diene was obtained according to the literature procedure (Wilcox *et al.*, 1961). First fraction from the final distillation of spiro[2.4]hepta-4,6-diene (2.05 g) was stored at room temperature for few weeks. After this time large, colorless crystals of the title compound deposited with 54% (1.10 g) yield.

### Refinement

All H atoms were positioned geometrically and refined using a riding model, with C–H = 0.93–0.98 Å, *U*_iso_(H) = 1.2 *U*_eq_(C).

### Crystal data

**Table:**

C_14_H_16_	*Z* = 2
*M**_r_* = 184.27	*F*(000) = 200
Triclinic, *P*1	*D*_x_ = 1.158 Mg m^−^^3^
Hall symbol: -P 1	Mo *K*α radiation, λ = 0.71073 Å
*a* = 6.4079 (5) Å	Cell parameters from 1384 reflections
*b* = 8.6355 (8) Å	θ = 2.6–28.5°
*c* = 10.7216 (10) Å	µ = 0.07 mm^−^^1^
α = 68.488 (9)°	*T* = 293 K
β = 81.625 (7)°	Block, colourless
γ = 73.351 (8)°	0.23 × 0.22 × 0.21 mm
*V* = 528.27 (8) Å^3^	

### Data collection

**Table:**

Oxford Diffraction Xcalibur S diffractometer	2269 independent reflections
graphite	1348 reflections with *I* > 2σ(*I*)
Detector resolution: 8.1883 pixels mm^-1^	*R*_int_ = 0.024
ω scans	θ_max_ = 27.0°, θ_min_ = 2.6°
Absorption correction: multi-scan (*CrysAlis PRO*; Oxford Diffraction, 2009)	*h* = −8→8
*T*_min_ = 0.775, *T*_max_ = 1	*k* = −10→10
3444 measured reflections	*l* = −8→13

### Refinement

**Table:**

Refinement on *F*^2^	Primary atom site location: structure-invariant direct methods
Least-squares matrix: full	Secondary atom site location: difference Fourier map
*R*[*F*^2^ > 2σ(*F*^2^)] = 0.060	Hydrogen site location: inferred from neighbouring sites
*wR*(*F*^2^) = 0.168	H-atom parameters constrained
*S* = 0.99	*w* = 1/[σ^2^(*F*_o_^2^) + (0.095*P*)^2^] where *P* = (*F*_o_^2^ + 2*F*_c_^2^)/3
2269 reflections	(Δ/σ)_max_ < 0.001
127 parameters	Δρ_max_ = 0.22 e Å^−^^3^
0 restraints	Δρ_min_ = −0.15 e Å^−^^3^

### Special details

**Table:**

Geometry. All e.s.d.'s (except the e.s.d. in the dihedral angle between two l.s. planes) are estimated using the full covariance matrix. The cell e.s.d.'s are taken into account individually in the estimation of e.s.d.'s in distances, angles and torsion angles; correlations between e.s.d.'s in cell parameters are only used when they are defined by crystal symmetry. An approximate (isotropic) treatment of cell e.s.d.'s is used for estimating e.s.d.'s involving l.s. planes.
Refinement. Refinement of *F*^2^ against ALL reflections. The weighted *R*-factor *wR* and goodness of fit *S* are based on *F*^2^, conventional *R*-factors *R* are based on *F*, with *F* set to zero for negative *F*^2^. The threshold expression of *F*^2^ > σ(*F*^2^) is used only for calculating *R*-factors(gt) *etc*. and is not relevant to the choice of reflections for refinement. *R*-factors based on *F*^2^ are statistically about twice as large as those based on *F*, and *R*- factors based on ALL data will be even larger.

### Fractional atomic coordinates and isotropic or equivalent isotropic displacement parameters (Å^2^)

**Table:**

	*x*	*y*	*z*	*U*_iso_*/*U*_eq_	
C1	0.7397 (3)	0.6967 (3)	0.2398 (2)	0.0523 (6)	
H1A	0.7789	0.7846	0.2623	0.063*	
C2	0.7772 (3)	0.5136 (3)	0.35036 (19)	0.0469 (5)	
H2A	0.7291	0.522	0.4392	0.056*	
C3	0.9992 (3)	0.3926 (3)	0.3543 (2)	0.0577 (6)	
H3A	1.1234	0.4104	0.3763	0.069*	
C4	0.9994 (3)	0.2591 (3)	0.3232 (2)	0.0539 (6)	
H4A	1.1241	0.1731	0.3191	0.065*	
C5	0.7809 (3)	0.2602 (2)	0.29543 (19)	0.0441 (5)	
C6	0.6293 (3)	0.4281 (2)	0.30671 (18)	0.0398 (5)	
H6A	0.5122	0.4035	0.3749	0.048*	
C7	0.5333 (3)	0.5718 (2)	0.17594 (18)	0.0451 (5)	
H7A	0.4042	0.5598	0.145	0.054*	
C8	0.7187 (4)	0.5983 (3)	0.0741 (2)	0.0559 (6)	
H8A	0.7437	0.5677	−0.0024	0.067*	
C9	0.8395 (3)	0.6717 (3)	0.1112 (2)	0.0594 (6)	
H9A	0.9646	0.7027	0.0658	0.071*	
C10	0.5010 (3)	0.7287 (2)	0.21729 (19)	0.0455 (5)	
C11	0.3131 (4)	0.7909 (3)	0.3016 (2)	0.0631 (6)	
H11A	0.3434	0.8275	0.371	0.076*	
H11B	0.1933	0.7363	0.3235	0.076*	
C12	0.3480 (4)	0.8997 (3)	0.1568 (2)	0.0646 (6)	
H12A	0.2489	0.9102	0.0921	0.078*	
H12B	0.399	1.0014	0.1396	0.078*	
C13	0.7460 (4)	0.1787 (3)	0.2006 (2)	0.0620 (6)	
H13A	0.6199	0.2352	0.1456	0.074*	
H13B	0.874	0.1232	0.1577	0.074*	
C14	0.7068 (4)	0.0959 (3)	0.3469 (2)	0.0644 (6)	
H14B	0.8112	−0.0099	0.393	0.077*	
H14C	0.5569	0.1022	0.3809	0.077*	

### Atomic displacement parameters (Å^2^)

**Table:**

	*U*^11^	*U*^22^	*U*^33^	*U*^12^	*U*^13^	*U*^23^
C1	0.0501 (12)	0.0406 (12)	0.0711 (14)	−0.0082 (9)	−0.0029 (10)	−0.0272 (11)
C2	0.0463 (11)	0.0487 (13)	0.0494 (11)	−0.0028 (9)	−0.0071 (9)	−0.0260 (10)
C3	0.0425 (12)	0.0604 (15)	0.0706 (14)	−0.0029 (10)	−0.0187 (10)	−0.0245 (12)
C4	0.0396 (11)	0.0502 (14)	0.0637 (13)	0.0054 (10)	−0.0080 (9)	−0.0207 (11)
C5	0.0454 (11)	0.0349 (11)	0.0479 (11)	−0.0001 (9)	−0.0058 (9)	−0.0156 (9)
C6	0.0368 (10)	0.0360 (11)	0.0435 (10)	−0.0049 (8)	0.0011 (8)	−0.0143 (8)
C7	0.0419 (10)	0.0398 (12)	0.0527 (12)	0.0024 (9)	−0.0124 (9)	−0.0205 (9)
C8	0.0647 (14)	0.0459 (13)	0.0417 (11)	0.0098 (11)	−0.0027 (10)	−0.0156 (10)
C9	0.0503 (13)	0.0419 (13)	0.0681 (14)	−0.0068 (10)	0.0119 (11)	−0.0077 (11)
C10	0.0434 (11)	0.0351 (12)	0.0542 (12)	0.0013 (9)	−0.0025 (9)	−0.0193 (9)
C11	0.0592 (14)	0.0509 (15)	0.0694 (15)	0.0054 (11)	0.0040 (11)	−0.0267 (12)
C12	0.0638 (14)	0.0429 (14)	0.0744 (16)	0.0062 (11)	−0.0035 (12)	−0.0204 (12)
C13	0.0758 (15)	0.0457 (14)	0.0672 (15)	−0.0036 (12)	−0.0123 (12)	−0.0279 (12)
C14	0.0731 (15)	0.0398 (13)	0.0736 (16)	−0.0078 (11)	−0.0065 (12)	−0.0158 (11)

### Geometric parameters (Å, °)

**Table:**

C1—C9	1.496 (3)	C7—C10	1.525 (2)
C1—C10	1.513 (3)	C7—H7A	0.98
C1—C2	1.566 (3)	C8—C9	1.309 (3)
C1—H1A	0.98	C8—H8A	0.93
C2—C3	1.500 (3)	C9—H9A	0.93
C2—C6	1.564 (2)	C10—C12	1.489 (3)
C2—H2A	0.98	C10—C11	1.491 (3)
C3—C4	1.312 (3)	C11—C12	1.514 (3)
C3—H3A	0.93	C11—H11A	0.97
C4—C5	1.470 (3)	C11—H11B	0.97
C4—H4A	0.93	C12—H12A	0.97
C5—C13	1.503 (3)	C12—H12B	0.97
C5—C14	1.509 (3)	C13—C14	1.483 (3)
C5—C6	1.532 (3)	C13—H13A	0.97
C6—C7	1.556 (3)	C13—H13B	0.97
C6—H6A	0.98	C14—H14B	0.97
C7—C8	1.500 (3)	C14—H14C	0.97
			
C9—C1—C10	100.07 (16)	C9—C8—C7	108.46 (17)
C9—C1—C2	106.78 (17)	C9—C8—H8A	125.8
C10—C1—C2	99.49 (14)	C7—C8—H8A	125.8
C9—C1—H1A	116	C8—C9—C1	107.59 (16)
C10—C1—H1A	116	C8—C9—H9A	126.2
C2—C1—H1A	116	C1—C9—H9A	126.2
C3—C2—C6	103.53 (15)	C12—C10—C11	61.07 (14)
C3—C2—C1	117.77 (17)	C12—C10—C1	125.94 (18)
C6—C2—C1	102.59 (14)	C11—C10—C1	126.01 (17)
C3—C2—H2A	110.8	C12—C10—C7	125.59 (17)
C6—C2—H2A	110.8	C11—C10—C7	125.14 (17)
C1—C2—H2A	110.8	C1—C10—C7	94.78 (15)
C4—C3—C2	112.80 (18)	C10—C11—C12	59.39 (13)
C4—C3—H3A	123.6	C10—C11—H11A	117.8
C2—C3—H3A	123.6	C12—C11—H11A	117.8
C3—C4—C5	112.61 (19)	C10—C11—H11B	117.8
C3—C4—H4A	123.7	C12—C11—H11B	117.8
C5—C4—H4A	123.7	H11A—C11—H11B	115
C4—C5—C13	122.29 (18)	C10—C12—C11	59.55 (14)
C4—C5—C14	120.29 (18)	C10—C12—H12A	117.8
C13—C5—C14	58.99 (13)	C11—C12—H12A	117.8
C4—C5—C6	105.79 (15)	C10—C12—H12B	117.8
C13—C5—C6	123.02 (17)	C11—C12—H12B	117.8
C14—C5—C6	120.92 (17)	H12A—C12—H12B	115
C5—C6—C7	118.11 (15)	C14—C13—C5	60.73 (13)
C5—C6—C2	105.17 (14)	C14—C13—H13A	117.7
C7—C6—C2	102.28 (14)	C5—C13—H13A	117.7
C5—C6—H6A	110.2	C14—C13—H13B	117.7
C7—C6—H6A	110.2	C5—C13—H13B	117.7
C2—C6—H6A	110.2	H13A—C13—H13B	114.8
C8—C7—C10	99.26 (15)	C13—C14—C5	60.28 (13)
C8—C7—C6	107.61 (16)	C13—C14—H14B	117.7
C10—C7—C6	99.27 (14)	C5—C14—H14B	117.7
C8—C7—H7A	116.1	C13—C14—H14C	117.7
C10—C7—H7A	116.1	C5—C14—H14C	117.7
C6—C7—H7A	116.1	H14B—C14—H14C	114.9
			
C9—C1—C2—C3	45.1 (2)	C6—C7—C8—C9	−70.4 (2)
C10—C1—C2—C3	148.69 (16)	C7—C8—C9—C1	0.2 (2)
C9—C1—C2—C6	−67.80 (18)	C10—C1—C9—C8	−33.2 (2)
C10—C1—C2—C6	35.83 (17)	C2—C1—C9—C8	70.0 (2)
C6—C2—C3—C4	−0.8 (2)	C9—C1—C10—C12	−91.6 (2)
C1—C2—C3—C4	−113.1 (2)	C2—C1—C10—C12	159.29 (19)
C2—C3—C4—C5	−1.3 (3)	C9—C1—C10—C11	−169.39 (19)
C3—C4—C5—C13	150.9 (2)	C2—C1—C10—C11	81.5 (2)
C3—C4—C5—C14	−138.8 (2)	C9—C1—C10—C7	49.82 (17)
C3—C4—C5—C6	2.8 (2)	C2—C1—C10—C7	−59.27 (16)
C4—C5—C6—C7	110.17 (18)	C8—C7—C10—C12	92.4 (2)
C13—C5—C6—C7	−37.6 (3)	C6—C7—C10—C12	−157.93 (19)
C14—C5—C6—C7	−108.5 (2)	C8—C7—C10—C11	169.4 (2)
C4—C5—C6—C2	−3.09 (19)	C6—C7—C10—C11	−80.9 (2)
C13—C5—C6—C2	−150.86 (18)	C8—C7—C10—C1	−49.28 (17)
C14—C5—C6—C2	138.22 (18)	C6—C7—C10—C1	60.43 (16)
C3—C2—C6—C5	2.37 (18)	C1—C10—C11—C12	115.3 (2)
C1—C2—C6—C5	125.38 (16)	C7—C10—C11—C12	−115.1 (2)
C3—C2—C6—C7	−121.60 (17)	C1—C10—C12—C11	−115.4 (2)
C1—C2—C6—C7	1.41 (17)	C7—C10—C12—C11	114.4 (2)
C5—C6—C7—C8	−49.8 (2)	C4—C5—C13—C14	108.4 (2)
C2—C6—C7—C8	65.04 (17)	C6—C5—C13—C14	−108.9 (2)
C5—C6—C7—C10	−152.68 (15)	C4—C5—C14—C13	−111.8 (2)
C2—C6—C7—C10	−37.84 (17)	C6—C5—C14—C13	112.4 (2)
C10—C7—C8—C9	32.5 (2)		
